# Double homozygous waltzer and Ames waltzer mice provide no evidence of retinal degeneration

**Published:** 2008-12-08

**Authors:** Zubair M. Ahmed, Sten Kjellstrom, Ricky J. L. Haywood-Watson, Ronald A. Bush, Lori L. Hampton, James F. Battey, Saima Riazuddin, Gregory Frolenkov, Paul A. Sieving, Thomas B. Friedman

**Affiliations:** 1 National Institute on Deafness and Other Communication Disorders, Bethesda, MD; 2 Molecular and Cellular Biology Program, Tulane University, New Orleans, LA; 3 National Institute of Neurological Disorders and Stroke, Bethesda, MD; 4 Department of Physiology, University of Kentucky, Lexington, KY; 5 National Eye Institute, National Institutes of Health, Bethesda, MD

## Abstract

**Purpose:**

To determine whether cadherin 23 and protocadherin 15 can substitute for one another in the maintenance of the retina and other tissues in the mouse. Does homozygosity for both *v* and *av* mutant alleles (i.e., a double homozygous mouse) cause retinal degeneration or an obvious retinal histopathology?

**Methods:**

We generated mice homozygous for both *Cdh23^v-6J^* and *Pcdh15^av-Jfb^* alleles. The retinal phenotypes of double heterozygous and double homozygous mutant mice were determined by light microscopy and electroretinography (ERG). Histology on 32 different tissues, scanning electron microscopy of organ of Corti hair cells as well as serum biochemical and hematological examinations were evaluated.

**Results:**

ERG waves of double heterozygous and double homozygous mice showed similar shape, growth of the amplitude with intensity, and implicit time for both rod and cone pathway mediated responses. Mice homozygous for both *Cdh23^v-6J^* and *Pcdh15^av-Jfb^* mutations showed no sign of retinitis pigmentosa or photoreceptor degeneration but, as expected, were deaf and had disorganized hair cell sensory bundles.

**Conclusions:**

The simultaneous presence of homozygous mutant alleles of cadherin 23 and protocadherin 15 results only in deafness, not retinal degeneration or any other additional obvious phenotype of the major organ systems. We conclude that in the mouse cadherin 23 or protocadherin 15 appear not to compensate for one another to maintain the retina.

## Introduction

Usher syndrome type 1 (USH1) in humans is a neurosensory disorder characterized by profound congenital deafness, vestibular areflexia, and progressive retinitis pigmentosa (RP) [1,2]. Seven USH1 loci have been reported [3,4]. Five USH1 genes have now been identified [1,5] and encode unconventional myosin VIIa (*MYO7A*), harmonin (*USH1C*), cadherin 23 (*CDH23*), protocadherin 15 (*PCDH15*), and sans (*SANS*) [6–13]. Although many of the mutant alleles of these genes cause USH some possibly hypomorphic alleles of *MYO7A, USH1C*, *CDH23,* and *PCDH15* are associated only with nonsyndromic hearing loss [6,10,14–16].

Mouse models of the USH1 genes (*Myo7a*, shaker 1; *Ush1c*, deaf circler; *Cdh23,* waltzer; *Pcdh15,* Ames waltzer; *Sans,* Jackson shaker) exhibit circling behavior, head tossing and profound sensorineural hearing loss, but do not exhibit retinal degeneration [17–23]. In the mammalian inner ear, myosin VIIa, harmonin, cadherin 23, protocadherin 15, and sans are expressed in hair cells. Some of these proteins interact in vitro or in heterologous expression systems, providing evidence of a macromolecular complex that is essential for the cohesiveness of stereocilia of sensory hair cells [24–28]. Protocadherin 15 and cadherin 23 are known to be constituents of links between stereocilia [14,29,30], including tip links [31,32] that are critical for normal mechanoelectrical transduction in hair cells [33].

In the retina, myosin VIIa, harmonin, cadherin 23, and protocadherin 15 isoform CD1 [31] are localized at photoreceptor synapses and expressed in photoreceptor cells, while myosin VIIa is also expressed in the apical processes of retinal pigmented epithelium [8,14,28,34,35]. Many of the USH1 proteins are also localized in the connecting cilium as well as in the periciliary protein network of photoreceptor cells, suggesting that the retinopathy component of USH may be thought of as a ciliopathy [23,26,35–39].

Some mouse models of USH do partially recapitulate the human visual phenotype. A knockout mouse model of human *USH2a* showed signs of a gradual loss of photoreceptors and an approximately 60% reduction in electroretinography (ERG) a-wave and b-wave amplitudes at 20 months of age [40]. Also, nine-month-old Jackson shaker (*Ush1c*) homozygous mutant mice have a mild peripheral photoreceptor degeneration, which is not accompanied by a reduction in ERG [21]. In contrast, and unlike humans, mice with disabling mutations of myosin VIIa, cadherin 23, protocadherin 15, or sans are reported to have normal histology of the retina [20].

The absence of an overt retinal phenotype in these mutant mice may reflect one or more differences between human and mouse in the functional requirements of the USH1 proteins in the retina, but not the inner ear. First, in the mouse, there may be functional redundancies for the USH1 proteins that do not occur in human retina [4,14,41]. Second, no reported *USH1* alleles in mice are precisely identical to the mutations of *MYO7A*, *USH1C*, *SANS*, *CDH23,* or *PCDH15* that cause USH1 in humans. A knockin mouse model homozygous for a precisely equivalent USH1 human mutation would permit a test of this possible, but unlikely, explanation for the absence of RP in mouse models of USH1. Third, mice have short life spans [42]; RP in USH patients detected by funduscopy is not evident for several years, although ERG evaluations of young USH1 patients do show abnormalities foreshadowing the onset of RP [43]. Fourth, there may be environmental factors, such as the intensity and duration of light exposure, that can have an impact on the onset and severity of RP. Experimental mice are nocturnal and usually live in dimly lit cages.

Protocadherin 15 and cadherin 23 are reported to physically interact with each other [32], and there is a report of digenic inheritance of USH1 involving heterozygous mutant alleles of both *CDH23* and *PCDH15* in humans and mice [44]. Therefore, we thought that it was possible that, unlike in the human retina, in the mouse retina there is functional redundancy between cadherin 23 and protocadherin 15. If functional redundancy is the explanation, we would expect a double homozygous mutant mouse (*Cdh23^v-6J^ Pcdh15^av-Jfb^*/*Cdh23^v-6J^ Pcdh15^av-Jfb^*) to exhibit retinal degeneration.

## Methods

### Breeding to generate *Cdh23^v-6J^* and *Pcdh15^av-Jfb^* double mutant mice

Animal procedures were conducted in accordance with the NIDCD/NIH Animal Care and Use Committee, protocol 1126–03. The *Pcdh15^av-Jfb^* mutant allele arose spontaneously in offspring segregating a gastrin-releasing peptide receptor (*Grpr*) null allele (B6.129-*Grpr^tm1Jfb^*) at the fifth backcross generation [45] and was maintained afterward by intercrossing animals. The *Cdh23^v-6J^* allele arose on the B10.A-*H2^h4^* (4R)/SgDvEg congenic strain at The Jackson Laboratory (Bar Harbor, ME). *Cdh23^v-6J^* mice were kindly provided by Konrad Noben-Trauth (NIDCD/NIH), which were then backcross-intercrossed with C57BL/6J mice for six generations.

*Pcdh15* is located 10 cM from *Cdh23* on mouse chromosome 10 [17]. To produce a chromosome with *Pcdh15^av-Jfb^* in cis to *Cdh23^v-6J^*, we crossed heterozygous *Pcdh15^av-Jfb^* females with homozygous *Cdh23^v-6J^* males. Double heterozygous F_1_ progeny (*Cdh23^v-6J^ +*/+ *Pcdh15^av-Jfb^*) were intercrossed. Mice homozygous for *Cdh23^v-6J^* and heterozygous for *Pcdh15^av-Jfb^* (*Cdh23^v-6J^* +/*Cdh23^v-6J^ Pcdh15^av-Jfb^*), which carry a chromosome with a crossover between *Cdh23^v-6J^* and *Pcdh15^av-Jfb^*, were identified. These mice were then crossed with C57BL/6J to obtain double cis-heterozygotes (*+ +*/*Cdh23^v-6J^ Pcdh15^av-Jfb^*). Intercrossing double cis-heterozygotes produced mice that were homozygous for both *Cdh23^v-6J^* and *Pcdh15^av-Jfb^*. All of the genotypic combinations had a C57BL/6J genetic background.

### Genotyping

Genomic DNA was isolated from tail biopsies (DNeasy Blood and Tissue Kit; Qiagen, Valencia, CA). The *Pcdh15^av-Jfb^* allele was genotyped by PCR amplification of exon 17 of *Pcdh15* (GenBank AF281899) using the forward primer 5′-GAT CTT CGC ATC CAA TGC AG-3′ and reverse primer 5′-AGC ATT TCG TTC TGG GTG AAT A-3′. Amplimers were sequence verified using Big DyeTerminator (ABI version 3.1; Applied Biosystems, Foster City, CA), size separated on a 3730xl DNA Analyzer (Applied Biosystems), and data analyzed using Lasergene (DNASTAR Inc.,

Madison, WI). The *Cdh23^v-6J^* mutation was genotyped by PCR amplification of exon 9 of *Cdh23* (GenBank AY026062) by using the forward primer 5′-AAA GGG CGC AGT AAT CTG TG-3′ and reverse primer 5′-TCC ACA CCT TCC AAG TAG GG-3′. Amplimers were sequence verified as we have described.

### Scanning electron microscopy

Scanning electron microscopy (SEM) examinations were performed on the organ of Corti. Mice were euthanized with CO_2_, decapitated, and their cochleae were removed and fixed in a solution containing 2.5% glutaraldehyde in 0.1 M cacodylate buffer supplemented with 2 mM CaCl_2_. To expedite fixation of the organ of Corti, we perfused the cochlea with the fixative via the holes at the apex and at the round window. This was followed incubation of cochlea in the same fixative for 1.5 h at room temperature. The organ of Corti was dissected from the modiolus in Hank's balanced salt solution (Invitrogen, Carlsbad, CA) and dehydrated with an acetone series. The specimens were critical point dried from liquid CO_2_ (Bal-Tec CPD030, Leica Microsystems, Wetzlar, Germany), sputter-coated (Balzers MED010, Balzers, Liechtensein) with platinum (5 nm, controlled by STM-100/MF film thickness monitor, Sycon Instruments, East Syracuse, NY), and observed with a field-emission scanning electron microscope (S-4500, Hitachi Technologies, Schaumburg, IL).

### Histological evaluation

Histology was performed on 32 different tissues from P120 male mice obtained from two wild-type (+ +/+ +), two cis-heterozygotes (*+ +*/*Cdh23^v-6J^ Pcdh15^av-Jfb^*), and two double homozygotes (*Cdh23^v-6J^ Pcdh15^av-Jfb^*/*Cdh23^v-6J^ Pcdh15^av-Jfb^*). Tissues examined included brain, thymus, spleen, pancreas, lymph nodes, liver, kidneys, adrenal gland, salivary glands, Harderian gland, trachea, thyroid, esophagus, aorta, lung, testes, epididymis, urinary bladder, ovaries, uterus, oviducts, cervix, prostate, seminal vesicles, preputial gland, heart, tongue, skeletal muscle, sciatic nerve, eyes, stomach, small intestine, cecum, colon, rectum, skin, sternum, vertebrae, femur, and spinal cord. Tissues were fixed in buffered aqueous zinc formalin, embedded in paraffin, sectioned, and stained with hematoxylin and eosin (HE) then examined with bright-field microscopy. Tissue samples containing bone were decalcified in a solution of formic acid and sodium citrate before sectioning.

To evaluate histological differences between wild-type, heterozygous, and double homozygous mutant mice retinas, we measured the outer nuclear layer (ONL) width at 200 µm intervals on midretinal sections beginning 200 µm from the center of the retina, close to the optic nerve head. We measured ONL width from the retinas of two wild-type, two heterozygous (*+ +*/*Cdh23^v-6J^ Pcdh15^av-Jfb^*), and two 4-month-old double homozygous (*Cdh23^v-6J^ Pcdh15^av-Jfb^*/*Cdh23^v-6J^ Pcdh15^av-Jfb^*) mutant mice. Eight measurements on each side of the central retina were made using digital photomicrographs taken with a 40X objective.

### Serum chemical and hematological analyses

Sera were obtained from six P120 male mice. There were two wild-types, two cis-heterozygotes (*+ +*/*Cdh23^v-6J^ Pcdh15^av-Jfb^*), and two double homozygotes (*Cdh23^v-6J^ Pcdh15^av-Jfb^*/*Cdh23^v-6J^ Pcdh15^av-Jfb^*), which were analyzed by the Department of Laboratory Medicine at the NIH for albumin, total bilirubin, total protein, alkaline phosphatase, ALT, AST, LDH, BUN, amylase, calcium, lipase, cholesterol, triglycerides, glucose, inorganic phosphorus, creatine kinase, gamma-glutamyl transpeptidase, and uric acid measurements. Hematological parameters measured were hemoglobin, white blood cell count, hematocrit, red blood cell count, mean corpuscular volume (MCV), and mean corpuscular hemoglobin concentration (MCHC). We used single-factor ANOVA to compare body to brain weight, spleen to brain weight, thymus to brain weight, heart to brain weight, liver to brain weight, and kidney to brain weight. No significant differences were found between mutant and controls (data not shown).

### Electroretinograms

Electroretinograms (ERGs) were recorded from one 5-month-old and one 9-month-old cis-heterozygote (+ *+*/*Cdh23^v-6J^ Pcdh15^av-Jfb^*), a 3-month-old and a 5-month-old homozygote for *Cdh23^v-6J^* that were also heterozygous for *Pcdh15^av-Jfb^* (*Cdh23^v-6J^* +/*Cdh23^v-6J^ Pcdh15^av-Jfb^*), and a 3-month-old, two 5-month-old and one 9-month-old double-homozygotes (*Cdh23^v-6J^ Pcdh15^av-Jfb^*/*Cdh23^v-6J^ Pcdh15^av-Jfb^*). Mice were dark-adapted for 12 h before they were anesthetized by an intraperitoneal injection of 80 mg/kg ketamine and 4 mg/kg xylazine. The pupils were dilated with topical 0.5% tropicamide and 0.5% phenylephrine HCl, and body temperature of the mice was maintained near 38 °C with a heating pad. ERGs were recorded simultaneously from both eyes. This was done by placing gold wire loops on the cornea with a drop of methylcellulose after administration of 1% proparacaine topical anesthetic. Gold wires were also put on the sclera at the limbus as the differential electrodes and a ground wire was attached to the left paw.

Rod pathway function was evaluated by eliciting scotopic ERG responses in the dark-adapted state using single xenon photostrobe flashes (PS33 Photopic Stimulator; Grass Instrument Co., West Warwick, RI) delivered in a Ganzfield light integrating sphere with inter-stimulus intervals of 3 to 60 s depending on stimulus intensity. Stimulus intensities in the range of −6.9 to +0.6 log cd-s/m^2^ were obtained using neutral density filters (Wratten, Eastman Kodak; Rochester, NY). Responses were amplified 5,000X at 0.1–1,000 Hz frequency band (3 dB cut points) using a 60 Hz line filter (CP511 AC amplifier, Grass Instrument Co).

Cone pathway function was evaluated by eliciting photopic responses in a light-adapted state on a rod-suppressing white background of 34 cd/m^2^ using single flashes at 2 s inter-stimulus intervals. For each intensity, 20 responses were averaged. A-waves were measured from the pre-stimulus baseline to the initial trough. B-waves were measured either from the baseline or from the a-wave trough when present. Implicit times were measured from flash onset to the a-wave maximum and the b-wave maximum.

### PCR analyses of inner ear and retinal cDNAs

Poly(A)^+^ RNA, isolated from P1-P5 inner ear and eye tissues of 50 C57BL/6J mice using Poly(A)Pure^TM^ mRNA reagents (Ambion, Austin, TX), and cDNA was prepared using an oligo dT primer and PowerScript^TM^ reverse transcriptase (Clontech, Palo Alto, CA). *Cdh23* (GenBank AY026062) splice variants were amplified using a forward primer (5′-ATC ACA CGG AAG GTG AAT ATC CAA G-3′) in exon 5 and a reverse primer (5′-CAG TCA AGG TGA AAC GCT GGA TAA G-3′) in exon 15. cDNAs were PCR amplified using LA-Taq (Takara Mirus, Madison, WI) by denaturing at 95 °C for 2 min, 30 cycles at 95 °C for 45 s, 60 °C for 30 s and 68 °C for 3 min followed by a 15 min extension at 72 °C. PCR products were cloned and fully sequenced.

## Results

### Physical and biochemical evaluation

Mice heterozygous for a mutant allele of *Cdh23* and *Pcdh15* (+ +/*Cdh23^v-6J^ Pcdh15^av-Jfb^*) are physically indistinguishable from their wild-type or single heterozygous littermates. By comparison, double homozygous mice (*Cdh23^v-6J^ Pcdh15^av-Jfb^*/*Cdh23^v-6J^ Pcdh15^av-Jfb^*) are hyperactive, exhibit head tossing and circling behavior and, as expected, are deaf. This phenotype was expected since homozygosity for either mutant allele alone (*Cdh23^v-6J^* or *Pcdh15^av-Jfb^*) results in congenital deafness and vestibular dysfunctions [17,18]. However, there was no additional pathophysiology in the double homozygous mice. With the exception of the inner ear, histological examinations of 32 tissues, a chemistry panel, and hematology tests of serum derived from two wild type, two double cis-heterozygotes (*+ +*/*Cdh23^v-6J^ Pcdh15^av-Jfb^*) and two double homozygous (*Cdh23^v-6J^ Pcdh15^av-Jfb^*/ *Cdh23^v-6J^ Pcdh15^av-Jfb^*) males revealed no pathological or biochemical abnormalities (data not shown).

### Phenotype of the eye

**Figure 1:**
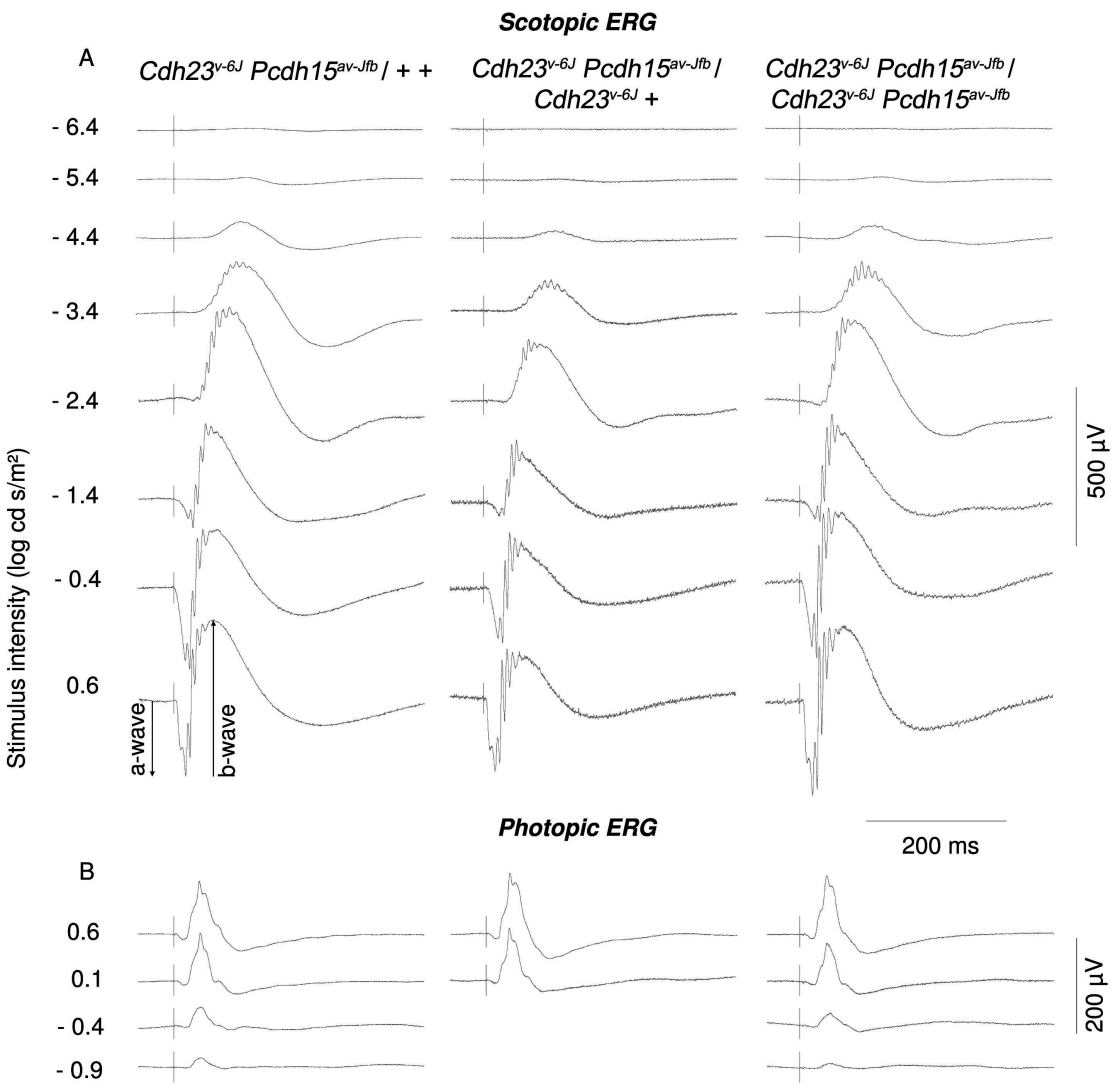
Electroretinogram waveforms recorded from 5-month-old mice. **A:** Double heterozygous mouse (*+ +*/*Cdh23^v-6J^ Pcdh15^av-Jfb^*), a *Cdh23^v-6J^* homozygote that is heterozygous for the *Pcdh15^av-Jfb^* allele (*Cdh23^v-6J^ +*/*Cdh23^v-6J^ Pcdh15^av-Jfb^*), and a double homozygous (*Cdh23^v-6J^ Pcdh15^av-Jfb^*/*Cdh23^v-6J^ Pcdh15^av-Jfb^*) mouse. Dark-adapted scotopic responses to the stimulus intensity range from −6.9 to +0.6 log cd-s/m^2^. **B:** Light-adapted photopic responses to the stimulus intensity range from −0.9 to +0.6 log cd-s/m^2^ on a 34 cd/m^2^ background.

To explore the effect of the mutations on retinal function, we compared ERGs from age-matched mutant mice since increasing age is correlated directly with a decline in b-wave amplitude [46]. Figure 1 shows waveforms from a group of four 5-month-old mice, that included one cis-heterozygote (+ +/*Cdh23^v-6J^ Pcdh15^av-Jfb^*), one *Cdh23^v-6J^* homozygote that was also a heterozygote for *Pcdh15^av-Jfb^* (*Cdh23^v-6J^*+/*Cdh23^v-6J^ Pcdh15^av-Jfb^*), and two double homozygotes (*Cdh23^v-6J^ Pcdh15^av-Jfb^*/*Cdh23^v-6J^ Pcdh15^av-Jfb^*). These waveforms showed comparable shape, growth of amplitude with intensity, and implicit time for both rod and cone pathway-mediated responses. The double homozygous line (*Cdh23^v-6J^ Pcdh15^av-Jfb^*/*Cdh23^v-6J^ Pcdh15^av-Jfb^*) and the double heterozygous line had similar amplitudes suggesting that there was no effect of the additional mutant alleles on retinal responses. The same outcome was observed for 3-month-old and 9-month-old double homozygous mice (data not shown).

**Figure 2:**
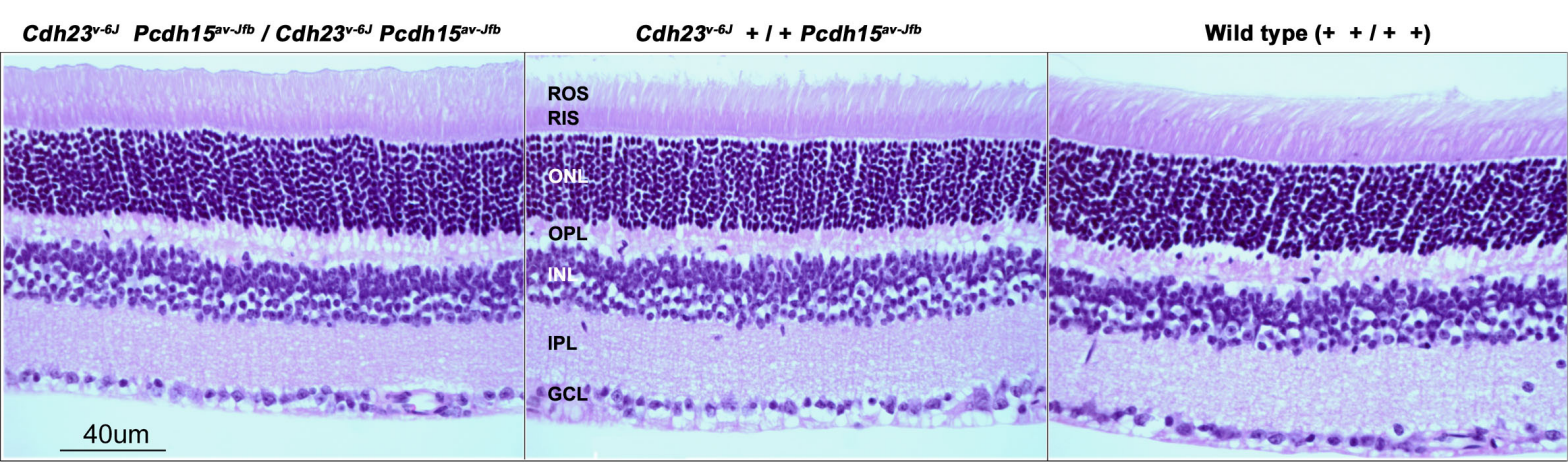
Light photomicrographs of retinal morphology of a wild-type, a double heterozygous (+ *+*/*Cdh23^v-6J^ Pcdh15^av-Jfb^*), and a double homozygous (*Cdh23^v-6J^ Pcdh15^av-Jfb^*/*Cdh23^v-6J^ Pcdh15^av-Jfb^*) mouse at P120. No structural abnormalities in the retinas of these mice were observed when compared to the wild-type mice. Images are of the mid-peripheral retina in sections taken close to the optic nerve of each eye. Abbreviations: rod outer segment (ROS); rod inner segments (RIS); outer nuclear layer (ONL); outer plexiform layer (OPL); inner nuclear layer (INL); inner plexiform layer (IPL); ganglion cell layer (GCL).

**Figure 3:**
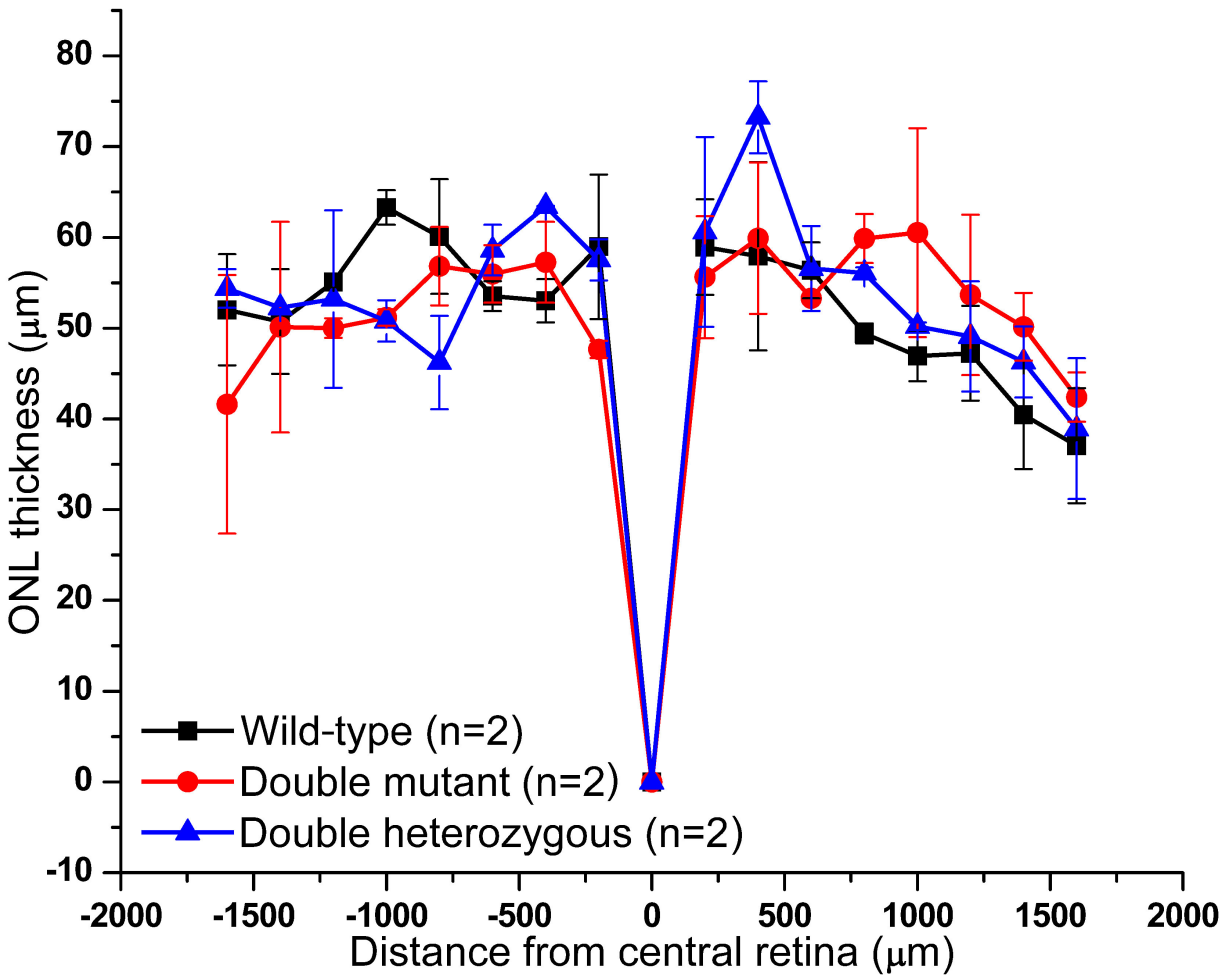
Measurements of outer nuclear layer in wild-type, heterozygous (*+ +*/*Cdh23^v-6J^ Pcdh15^av-Jfb^*), and double homozygous (*Cdh23^v-6J^* *Pcdh15^av-Jfb^*/*Cdh23^v-6J^ Pcdh15^av-Jfb^*) mice. Measurements were performed every 200 µm from central retina close to the optic nerve head to an area near the peripheral edge of retinal sections. One-way ANOVA of mean outer nuclear layer (ONL) width measurements indicate no significant effect of double homozygosity for *av* and *v* mutant alleles.

We next examined retinal morphology of 5-month-old wild-type (n=2), double heterozygous (+ *+*/*Cdh23^v-6J^ Pcdh15^av-Jfb^*; n=2) and double homozygous (*Cdh23^v-6J^ Pcdh15^av-Jfb^*/*Cdh23^v-6J^ Pcdh15^av-Jfb^*; n=2) mice. A portion of representative sections taken from the same region of a retina of each of these genotypes is shown in Figure 2. The width of the ONL was measured at 16 points in sections of the mouse retina from wild-type (mean 52.57 µm; SD 0.76), double heterozygotes (mean 54.21 µm; SD 0.03), and double homozygotes for *Cdh23^v-6J^* and *Pcdh15^av-Jfb^* mutant alleles (mean 52.88 µm; SD 0.76; Figure 3). We found no statistical difference (one-way ANOVA, ad hoc Bonferroni, SPSS 16 for Windows, SPSS, Chicago, IL) between the three genotypes.

### Hair cell stereocilia bundles

**Figure 4:**
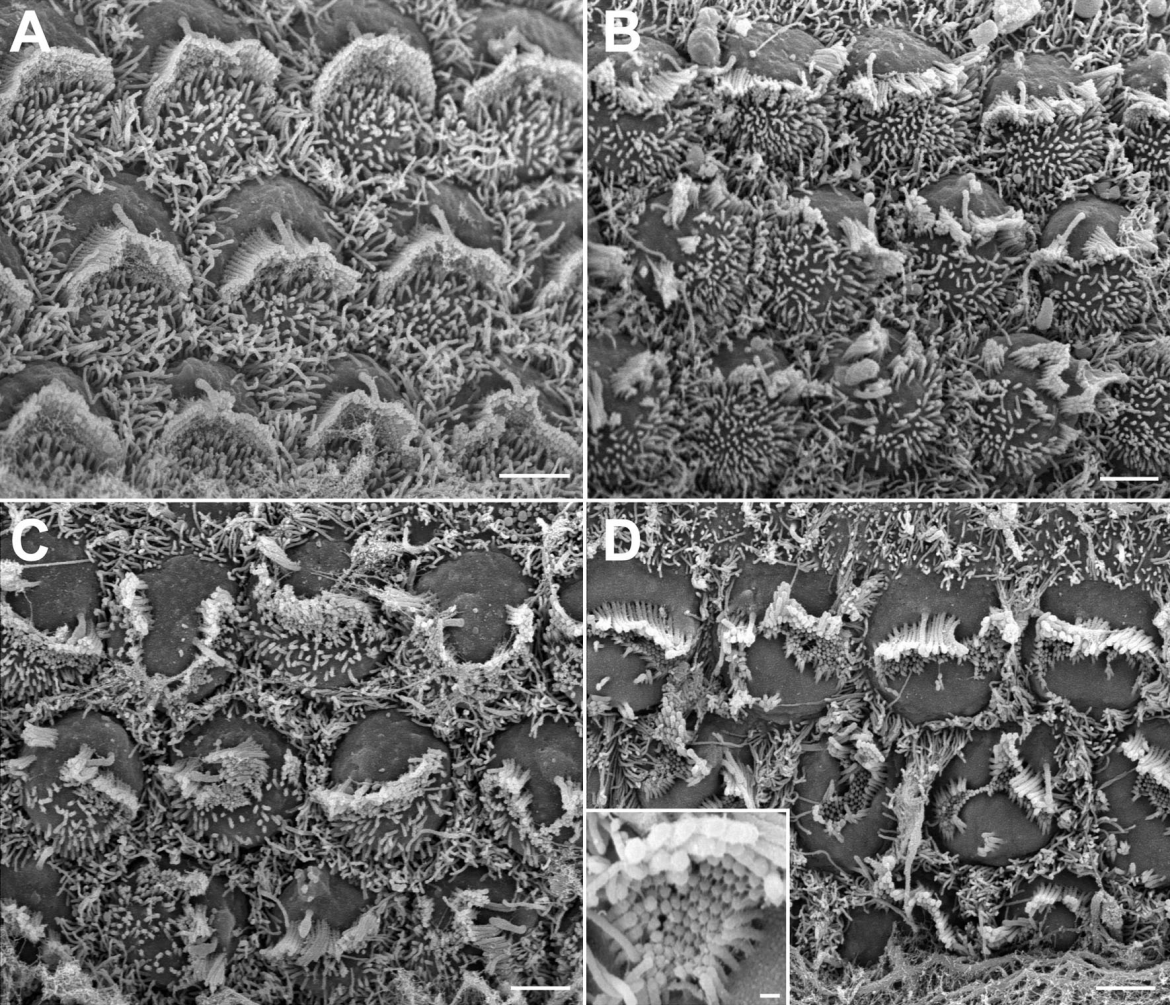
Scanning electron micrographs of the outer hair cells from the middle cochlear turn of **A:** P2 *Cdh23^v-6J^* heterozygote (*+ +*/*Cdh23^v-6J^* +), **B:** *Cdh23^v-6J^ +*/*Cdh23^v-6J^ Pcdh15^av-Jfb^* mouse, **C:** *+* *Pcdh15^av-Jfb^*/*Cdh23^v-6J^ Pcdh15^av-Jfb^* mouse, and **D:** *Cdh23^v-6J^ Pcdh15^av-Jfb^*/*Cdh23^v-6J^ Pcdh15^av-Jfb^* mouse. Inset shows a close-up view of a stereocilia bundle of a *Cdh23^v-6J^ Pcdh15^av-Jfb^*/*Cdh23^v-6J^ Pcdh15^av-Jfb^* mouse. Note the presence of stereocilia links. Scale bars in **A**-**D** equal 2 μm; scale bar in inset in **D** equals 200 nm.

The most prominent histopathological feature observed in the organ of Corti of neonatal (P2) double homozygous mice (*Cdh23^v-6J^ Pcdh15^av-Jfb^*/*Cdh23^v-6J^ Pcdh15^av-Jfb^*) is the disruption of the normally compact inner ear hair cell stereocilia bundle morphology (Figure 4). Generally, the hair cell phenotype of mice homozygous for *Cdh23^v-6J^* (Figure 4B) is somewhat less severe than mice homozygous for the *Pcdh15^av-Jfb^* allele (Figure 4C). Hair cell phenotypes of *Cdh23^v-6J^* and *Pcdh15^av-Jfb^* single homozygotes were not modified by the presence of one additional mutant allele of *Pcdh15* or *Cdh23,* respectively, as evaluated by SEM (Figure 4). However, the abnormal hair cell phenotypic features were additive in the double homozygotes (Figure 4D). The apical surface lacked microvilli, the stereocilia bundles were displaced, and the kinocilia were misplaced. In addition, the staircase architecture was also disrupted (Figure 4D), but some links between stereocilia were still present (Figure 4D, inset).

### Splice variant of *Cdh23*

**Figure 5:**
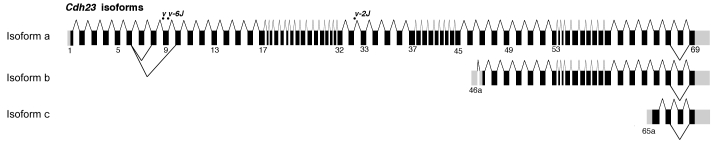
Schematic representation of the *Cdh23* gene structure and splice variants. Isoform a of *Cdh23* is the full-length transcript and contains 69 coding (black) and noncoding (gray) exons [18]. Isoforms b and c were previously reported [29]. Exon 68 encodes part of the cytoplasmic domain of cadherin 23 and is alternatively spliced. Two alternatively splice variants of isoform a, involving exons 5–10 (GenBank EU681829 and EU681830) of *Cdh23* were amplified from inner ear and retina cDNA using PCR primers located in the sequence of exons 5 and 13. *Cdh23^v-6J^* is a nonsense mutation found in exon 9, which is absent in one of the alternative splice variants of *Cdh23* isoform a.

The *Cdh23^v-6J^* allele is a G>T transversion (c.904G>T), which introduces a stop codon (p.E302X) in exon 9 [18]. To determine if there were inframe splice variants of *Cdh23* that did not include exon 9, we used a forward primer in exon 5 and reverse primer in exon 13 and PCR amplified inner ear and retina cDNA of wild-type and *Cdh23^v-6J^* mice. In addition to the reported transcript [18], there is an alternative splice variant of *Cdh23* that does not include exons 7 to 9 (Figure 5; GenBank EU681829 and EU681830). We previously reported multiple shorter isoforms from mouse inner ear and retina that do not include exons 1–39 of *Cdh23* (Figure 5; isoforms b, c) [29]. Thus the Ames waltzer *Cdh23^v-6J^* allele is not deficient for all isoforms of *Cdh23* but null only for isoforms that include exon 9.

## Discussion

USH1 is inherited as a simple Mendelian recessive trait, although there is considerable locus and allelic heterogeneity. A large number of pathogenic recessive mutant alleles of *CDH23* have been described that are associated with USH1 or nonsyndromic deafness DFNB12 [47]. To date, 21 recessive alleles of *PCDH15* at the *USH1F* locus and two mutant *DFNB23* alleles have been reported [14,47]. From our examination of the published data from USH segregating in multiplex families, we conclude that there is no evidence for digenic inheritance for this disorder. Digenic inheritance was reported as an explanation for three USH1 singeltons [44]. Each affected individual was found to be carrying one recessive mutant allele of *PCDH15* and one recessive mutant allele of *CDH23* [44], and it is the combination of these two nonallelic mutations that was presumed to cause type USH1 [44]. The authors [44] used SSCP analyses to screen for mutations of exons of *PCDH15* and *CDH23*, rather than direct DNA sequencing of all exons. However, not all of the exons of *PCDH15* [31,48] and *CDH23* [29] were known in 2005. An explanation for the report of digenic inheritance of two USH1 singeltons [44] is that one additional pathogenic allele in either *PCDH15* or *CDH23* was overlooked in SSCP screens for variants responsible for USH1. One of the three USH1 digenic subjects (family 1677) was reported to be homozygous for a pathogenic mutation (p.T1209A) of *CDH23,* a carrier of c.5601delAAC in *PCDH15* [44], and thus this individual is not an example of digenic inheritance of USH1. The authors suggested that the additional pathogenic mutation of *PCDH15* may have enhanced the severity of USH1. Although this idea is plausible, USH1-affected individuals show a surprisingly high degree of phenotypic variability for the severity of this disorder within and between families [49,50]. There is not yet a second corroborative report of digenic inheritance of USH1 [47].

During differentiation and maturation of the mouse inner ear, cadherin 23 and some isoforms of protocadherin 15 are expressed along the length of hair cell stereocilia [25,29–31]. As the stereocilia bundles mature, cadherin 23 staining becomes less prominent and after P16, cadherin 23 staining in stereocilia of both the organ of Corti and the vestibular organs of mouse and rat are at low levels [32], or perhaps absent [25,29,30]. In contrast, protocadherin 15 is easily detected in the stereocilia bundles of inner ear hair cells of even a 1-year-old mouse [14,29–31]. We demonstrated that protocadherin 15 is the tip link antigen (TLA) [31,51]. Thus protocadherin 15 is a component of the hair cell stereocilia tip link complex [31]. Kazmierczak et al. [32] provided evidence that protocadherin 15 and cadherin 23 physically interact and can form antiparallel heteromultimers.

Scanning EM analyses of homozygous *Cdh23^v-6J^* mice revealed a less severe inner ear hair cell stereocilia phenotype as compared to homozygous *Pcdh15^av-Jfb^* mice (Figure 4B,C). Mice homozygous for both *Cdh23^v-6J^* and *Pcdh15^av-Jfb^* had no obvious additional abnormalities but did show the combination of alterations associated with being homozygous for either a *Cdh23* or a *Pcdh15* mutant allele.

We next tested whether or not cadherin 23 or protocadherin 15 compensate for one another in the mouse retina by generating mice homozygous for mutant alleles of *Cdh23^v-6J^* and *Pcdh15^av-Jfb^*. We did not observe any retinal histopathology in *Cdh23^v-6J^ Pcdh15^av-Jfb^* double homozygous mutant mice at P120 (Figure 2). Moreover, the ERG a- and b- wave amplitudes and implicit times of homozygous double mutant mice were comparable with control heterozygous mice. The double heterozygous mice (*Cdh23^v-6J^ +*/+ *Pcdh15^av-Jfb^*) in our study also showed no obvious retinal abnormalities and thus these data provide no evidence for digenic inheritance.

In our previous study [41], we found a consistent reduction in a- and b-wave amplitudes of about 40% in homozygous compared to heterozygous *Pcdh15^av-Jfb^* and *Pcdh15^av-5J^* mutant mice at several ages. However, these mice did not show histopathology of the retina. Measurements of retina sections revealed no significant differences in either the ONL width or the rod outer segment (ROS) length as a function of genotype [41]. Similarly, in this study we did not observe retinal histopathology in 9-month-old *Cdh23^v-6J^ Pcdh15^av-Jfb^* double homozygous mutant mice. Consistent with these observations, the shaker 1, waltzer, Ames waltzer, and Jackson shaker mouse models of USH1 do not exhibit progressive retinal degeneration [1,41]. However, *Ush2a* knockout mice lacking usherin do show convincing retinal degeneration by 20 months of age [40]. It will be of interest to determine if 20-month-old double homozygous *Cdh23^v-6J^ Pcdh15^av-Jfb^* mice show retinal degeneration in excess of wild type control mice littermates.

As compared to human USH1 subjects, the lack of retinal degeneration in the single homozygous or in the double homozygous mutant mice could be due to functional redundancy with yet some other adhesion protein in the retina. We considered another possible explanation for the absence of retinal degeneration in mice homozygous for mutations of *Pcdh15* or *Cdh23*. The *Pcdh15^av-Jfb^* phenotype is caused by a single nucleotide insertion (c.2099insC) in exon 17, which causes a frameshift and introduces a premature stop codon in exon 18 [52]. If there were no alternative RNA splicing that excludes exon 17, then a mutant protocadherin 15, if synthesized, would be truncated after the sixth extracellular cadherin ectodomain. We previously reported multiple isoforms of *Pcdh15* from mouse inner ear and retina that alternatively include or exclude exon 17 [31]. These isoforms were also found in the inner ear and retina cDNA libraries derived from *Pcdh15^av-Jfb^* homozygotes (data not shown). In addition, we also found isoforms of *Pcdh15*, which circumvent the mutant alleles of *Pcdh15^av-J^*, *Pcdh15^av-2J^*, *Pcdh15^av-Tg^*, *Pcdh15^av-3J^*, and *Pcdh15^av-5J^* [31]. Thus, to date, all of the Ames waltzer alleles of *Pcdh15* are not null alleles of all isoforms of *Pcdh15*, but rather are just missing one or more but not all alternative splice isoforms [31]. However, all of these isoforms are necessary for inner ear function but not for the maintenance of the mouse retina [41]. In the present study, we report that exon 9 of *Cdh23*, the exon which harbors the *v^6J^* mutant allele, is also alternatively spliced. Perhaps one of the remaining isoforms of each of these two genes unaffected by the *v* and *av* alleles can maintain retinal function under ambient light conditions [31,41], but cannot maintain normal auditory function. Should this explanation prove to be correct for the absence of retinal degeneration in *v* and *av* mutant mice, then increasing the levels of particular isoforms of *CDH23* and *PCDH15* in the mutant human retina might delay the onset or perhaps retard the progress of the RP component of USH1.
